# Low Weight Polysaccharide of *Hericium erinaceus* Ameliorates Colitis via Inhibiting the NLRP3 Inflammasome Activation in Association with Gut Microbiota Modulation

**DOI:** 10.3390/nu15030739

**Published:** 2023-02-01

**Authors:** Yilin Ren, Qige Sun, Ruonan Gao, Yinyue Sheng, Tianyue Guan, Wang Li, Lingxi Zhou, Chang Liu, Huaxiang Li, Zhenming Lu, Lihua Yu, Jinsong Shi, Zhenghong Xu, Yuzheng Xue, Yan Geng

**Affiliations:** 1 Department of Gastroenterology, Affiliated Hospital of Jiangnan University, Wuxi 214122, China; 2 School of Medicine, Jiangnan University, Wuxi 214122, China; 3 Key Laboratory of Industrial Biotechnology of Ministry of Education, School of Biotechnology, Jiangnan University, Wuxi 214122, China; 4 School of Life Science and Health Engineering, Jiangnan University, Wuxi 214122, China; 5 School of Food Science and Technology, Jiangnan University, No. 1800 Lihu Avenue, Wuxi 214122, China; 6 College of Food Science and Engineering, Yangzhou University, Yangzhou 225127, China; 7 National Engineering Research Center for Cereal Fermentation and Food Biomanufacturing, Jiangnan University, Wuxi 214122, China

**Keywords:** polysaccharide, ulcerative colitis, NLRP3, *Hericium erinaceus*, predictive modeling

## Abstract

Ulcerative colitis (UC), one of the typical inflammatory bowel diseases caused by dysregulated immunity, still requires novel therapeutic medicine with high efficacy and low toxicity. *Hericium erinaceus* has been widely used to treat different health problems especially gastrointestinal sickness in China for thousands of years. Here, we isolated, purified, and characterized a novel low weight polysaccharide (HEP10, Mw: 9.9 kDa) from the mycelia of *H. erinaceus* in submerged culture. We explored the therapeutic effect of HEP10 on UC and explored its underlying mechanisms. On one hand, HEP10 suppressed the production of TNF-α, IL-1β, IL-6, inducible iNOS, and COX-2 in LPS challenged murine macrophage RAW264.7 cells, as well as in colons from DSS-induced colitis mice. On the other hand, HEP10 treatment markedly suppressed the activation of NLRP3 inflammasome, NF-κB, AKT, and MAPK pathways. Moreover, HEP10 reversed DSS-induced alternation of the gut community composition and structure by significantly increasing *Akkermansia muciniphila* and also promoting functional shifts in gut microbiota. Structural equation modeling also highlighted that HEP10 can change widely through gut microbiota. In conclusion, HEP10 has a better prebiotic effect than the crude polysaccharides of *H. erinaceus*, which can be used as a novel dietary supplement and prebiotic to ameliorate colitis.

## 1. Introduction

Ulcerative colitis (UC) is a type of inflammatory bowel disease (IBD), which is caused by dysregulated immunity with unknown etiology [1]. So far, inflammation and oxidative stress are known to play pivotal roles in the pathophysiology of UC [2]. Inflammasomes are multi-protein signaling platforms that play a critical role in innate immunity and inflammation. The activation of inflammasomes results in the activation of caspase-1 (CASP1) and a subsequent cleavage of proinflammatory cytokines interleukin (IL)-1β and IL-18, which facilitates the innate immune system fighting against pathogenic microbes and host-derived signals of cellular distress [3]. The NOD-like receptor family pyrin domain containing 3 (NLRP3) complex is the best characterized inflammasome complex, which contains NLRP3, ASC, phospho (p)-CASP1, and NIMA-related kinase 7 (NEK7) [4]. NLRP3 inflammasome is activated in a two-step process. The first step is the activation of NF-κB signaling, resulting in inflammasome initiation including NLRP3, pro-IL-1β, and pro-IL-18. The second step is the activation of NLRP3 inflammasome assembly and inflammatory reaction, leading to activation of CASP1 [3]. Emerging evidence suggests that NLRP3 inflammasome activation plays an essential role in the development and pathogenesis of UC [5,6].

Polysaccharides from functional food have been demonstrated to have multiple pharmacological activities with fewer side effects. Recently, polysaccharides have been considered as promising candidates to treat ulcerative colitis [7,8]. *Hericium erinaceus* (Higher Basidiomycetes, also known as Lion’s Mane Mushroom or Hedgehog Mushroom) is an edible and medicinal mushroom that belongs to the order Aphyllophorales and family Hydnaceae. *H. erinaceus* contains a variety of trace elements, proteins, unsaturated fatty acids, and carbohydrates, and it has been used in folk medicine in China to treat certain diseases including cancer, neurasthenia, and gastrointestinal ulcers [9,10]. A number of polysaccharides have been isolated from *H. erinaceus*, and they possess multiple beneficial functions, such as a hepatoprotective effect and anti-tumor, antioxidant, anti-gastritis, and immunomodulatory activities [10,11,12]. In our previous study, the crude polysaccharide from *H. erinaceus* mycelia (HECP) in submerged culture protected mice against inflammatory bowel disease induced by 2% dextran sulphate sodium (DSS) [7]. Thus, it is of interest to elucidate the structures and bioactivities of the purified polysaccharides in HECP. In this study, we isolated, purified, and characterized a low weight polysaccharide (HEP10, Mw: 9.9 kDa) from HECP. Then, we further investigated the role of HEP10 in NLRP3 inflammasome activation in vivo and in vitro. Furthermore, we deeply analyzed the role of HEP10 in modulating the structure and metabolic function of the gut microbiota.

## 2. Materials and Methods

### 2.1. Materials

Jiangsu Shenhua Pharmaceutical Co., Ltd. (Huaian, China) provided HECP for this study. Dextran sulfate sodium (DSS, 40 kD) was purchased from Shanghai Macklin Biochemical Technology Co., Ltd. (Shanghai, China).

### 2.2. Preparation and Purification of HEP10

HECP was prepared as previously described [7] and dissolved in distilled water. As shown in Figure S1, HECP was deproteinized using the Sevage method and then separated using an anion exchange column (DEAE-Sepharose Fast Flow, Φ 16 × 100 mm) and Sepharose G-75 chromatographic column (Φ 10 mm × 300 mm, GE Healthcare Bio-Sciences AB, Uppsala, Sweden). The columns were eluted with distilled water (4 mL/min) and NaCl solution (1 mL/min). The detection wavelength was 202 nm at 4 °C. The elution was dialyzed in distilled water, then freeze dried for subsequent experiments. The yield of HEP10 was 12.32%.

### 2.3. Structural Characterization of HEP10

#### 2.3.1. Sugar and Protein Contents

Total sugar content was detected by the standard phenol-sulfuric acid method [13]. The protein content was investigated by phenolic phenol kit (Shanghai Biomedical Engineering Co., Ltd., Shanghai, China).

#### 2.3.2. Monosaccharide Composition Analysis

*H. erinaceus* polysaccharide was hydrolyzed with 2 M trifluoroacetic acid at 110 °C for 4 h. The solution was concentrated and then washed with Methanol three times. After vacuum drying, the residue was dissolved in deionized water and analyzed with a CarboPac PA20 column through a high performance anion exchange chromatography (HPAEC) system (Dionex Corp, Sunnyvale, CA, USA) [14].

#### 2.3.3. Molecular Weight Analysis

The molecular weight of *H. erinaceus* polysaccharide was determined by High-performance Gel Permeation Chromatography (HPGPC, 6000 high performance liquid chromatograph, Waters, MA, USA). A 300 mm × 7.8 mmid × 2 Ultrahydrogel™ Linear (Waters, Milford, MA, USA) was eluted with 0.1 M NaNO_3_ (0.9 mL/min, 45 °C) and determined by a Waters 2410 RI detector. Calibrating the average molecular weight was performed using a pullulan polysaccharide calibration kit (Agilent Technologies, Santa Clara, CA, USA).

#### 2.3.4. Infrared (IR) Spectrometry

The freeze-dried HEP10 powder was evenly ground and mixed with KBr powder. The mixture was pressed into a 1 mm pellet and analyzed using a Nexus SDXC FT-IR spectrophotometer (Nicolet, Boston, MA, USA) within 4000–400 cm^−1^ at RT.

#### 2.3.5. NMR Spectroscopy Analysis

About 60 mg HEP10 powder was dissolved in 1 mL D_2_O and freeze dried twice. Then the residue was dissolved in 600 µL D_2_O and analyzed by AVANCE III 400 MHz NMR spectrometer (Bruker, Karlsruhe, Germany).

### 2.4. Animals

C57BL/6 mice (six-week-old male, SCXK (Hu) 2012–002) were acclimated for 7 days. All mice were free to drink tap water and were provided with a chow diet (M01-F25). The mice and diet were both provided from Shanghai SLAC Laboratories Animal Co., Shanghai, China. Mice were kept in a 25 °C room under conditions of 12 h of daylight and 12 h of darkness.

#### 2.4.1. Acute Colitis Model and HEP10 Treatment

**Figure 1:**
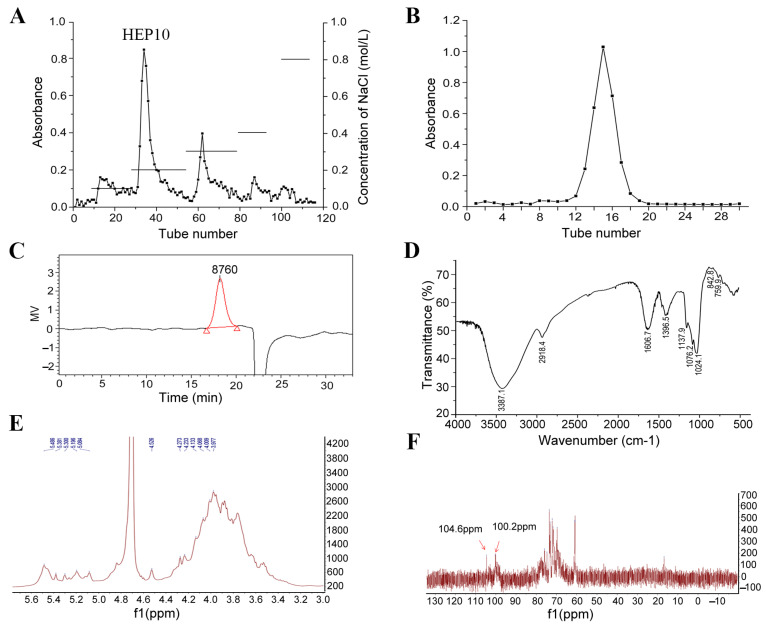
Structural characterization of HEP10. Chromatograms of HEP10 from (**A**) DEAE fast flow chromatography, (**B**) Superdex G-75 chromatography. (**C**) Molecular distribution of HEP10 by HPGPC. (**D**) FT-IR spectrum of HEP10. NMR spectra of HEP10: (**E**) ^1^H NMR spectrum; (**F**) ^13^C NMR spectrum.

The 25 mice were divided randomly into five groups. DSS (2% *w*/*v*) was administered ad libitum for 7 days to induce acute colitis in mice [15]. As showed in Figure 1A, the control (CTL) group received water. HEP10 treatment groups were administrated 50, 100, or 200 mg/kg HEP10 by gavage from day 0 to day 7. At day 8, mice were euthanized, and colons were collected immediately. In order to measure the severity of colitis, the disease activity index (DAI) was calculated by rectal bleeding, body weight loss (%), and stool consistency [15]. Distal colon parts were fixed in 4% PFA in PBS, embedded in paraffin, cut in 4 μm sections, and stained with Hematoxylin and eosin (H&E).

#### 2.4.2. Measurement of Nitric Oxide (NO) in Serum

In order to collect the serums, the blood samples were centrifuged for 15 min at 4 °C and 3000 rpm. According to the manufacturer’s protocol, NO content in serum was determined using a Jiancheng Bioengineering Institute’s assay kit (Nanjing, China).

#### 2.4.3. Assay of Colonic Antioxidant Index and Inflammatory Cytokines

Colonic tissue was homogenized in 50 mM ice-cold PBS solution. The MPO, T-SOD, and MDA activity assay kits were used to determine antioxidant indexes (Jiancheng Bioengineering Institute, Nanjing, China). IL-1β, IL-6, and tumor necrosis factor-α (TNF-α) cytokines were quantified using eBioscience ELISA kits following the manufacturer’s instructions (San Diego, CA, USA).

### 2.5. Cell Culture

From Fuxiang Central Experiment Laboratory of Fudan University, we obtained RAW264.7 mouse macrophage cell lines. RAW264.7 cells were routinely cultured in DMEM supplemented with 10% fetal bovine serum and 100 U/mL penicillin-streptomycin (ThermoFisher Scientific, Carlsbad, CA, USA) at 37 °C in a humidified atmosphere of 5% CO_2_.

#### 2.5.1. Cell Viability

2 × 10 ^5^ cells/mL cells were seeded in 96-well plates at 70–90% confluence. The cells were treated with HEP10 and/or lipopolysaccharide (LPS) for 24 h, and then MTT was added to each well and incubated for 4 h at 37 °C in the dark. After medium was aspirated, the blue formazan product obtained was dissolved in DMSO for 15 min. The absorbance at 570 nm was measured with a Micro-Reader (Thermolab Systems, Franklin, MA, USA). Cell viability (% of the control) of RAW264.7 cells was calculated as 100 × (absorbance of treated/absorbance of control).

#### 2.5.2. Measurement of NO/Nitrite and Cytokines in Culture Medium [16]

Briefly, the cultured media was mixed with the Griess reagent and incubated for 10 min at room temperature (RT). A Micro-Reader (Thermolab Systems, Franklin, MA, USA) was then used to measure the absorbance at 540 nm. Cytokines including TNF-α, IL-1β, and IL-6 were detected in the culture medium using standard ELISA method.

### 2.6. Quantitative Real-Time Polymerase Chain Reaction (qRT-PCR)

Total RNA was isolated from colon tissues by using the TRIzol reagent. qRT-PCR was performed according to the previous protocol [7]. Sequences of the specific primer sets are as follows: *Tnf-α* (NM_013693.2), forward, 5′-ccctcacactcagatcatcttct-3′, reverse, 5′-gctacgacgtgggctacag-3′; *Il-1β* (NM_008361), forward, 5′-ttgaagaagagcccatcctc-3′, reverse, 3′-cagctcatatgggtccgac-3′; *Il-6* (NM_031168), forward, 5′-tagtccttcctaccccaatttcc-3′, reverse, 5′-ttggtccttagccactccttc-3′. Glyceraldehyde-3-phosphate dehydrogenase (gapdh) was used as a control. The relative gene expressions were analyzed using 2^−ΔΔCT^ method.

### 2.7. Western Blot Analysis

Total proteins were obtained by homogenizing colon tissue or lysating cells in ice-cold RIPA buffer with protease inhibitor (Thermo Fisher Scientific, Rockford, IL, USA). The proteins were separated by 10% SDS-PAGE, then transferred onto PVDF membranes. After blocking with 5% *w*/*v* nonfat milk diluted in TBS-T buffer for 1 h, the membranes were incubated overnight with primary antibodies at 4 °C. NLRP3, ASC, CASP1, NF-κB p65 (p65), phospho-p65 (p-p65), inhibitor of NFkappa B-alpha (IκBα), p-IκBα, Protein kinase B (Akt), p-Akt, extracellular signal-regulated kinase (ERK), p-ERK, p38 MAPK (p38), p-p38, c-Jun N-terminal kinase (JNK), and p-JNK were purchased from Cell Signaling Technology (Boston, MA, USA). Cyclooxygenase-2 (COX-2), inducible nitric oxide synthase (iNOS), and GAPDH antibodies were purchased from Abcam (Cambridge, UK). The membranes were washed 3 times. Then, they were incubated with corresponding secondary antibodies for 1 h at RT. To quantify the immunoreactive bands, we used a Bio-Rad enhanced chemiluminescence system and Image J software to visualize the immunoreactive bands (Laboratories, San Francisco, CA, USA).

### 2.8. Gut Microbiota Analyses

Genomic DNA was isolated from caecal content using the QIAamp DNA Stool Mini Kit (QIAGEN, Fredrick, MD, USA). Two samples from the same group were randomly mixed with equal concentration and amount before 16S rDNA sequencing. 16S rDNA sequencing was conducted as previously described [7]. Briefly, the V3-V4 region of the bacteria 16S ribosomal RNA gene was amplified, then the sequencing was performed using an Illumina MiSeq. Reads were then processed using the QIIME (version 1.9.1, Flagstaff, AZ 86011, USA) analysis pipeline as described. OTU clustering was defined at the 97% similarity level. Taxonomy was assigned to all OTUs by searching against the version Aug, 2013 Greengenes Database. The heatmap was generated by the R software (version 3.6) [7] with the gplots package. Predicted functions of gut microbiota were analyzed by Phylogenetic Investigation of Communities by Reconstruction of Unobserved States (PICRUSt). The structural equation modeling was conducted as previously described [17].

### 2.9. Short Chain Fatty Acids (SCFAs) Analysis

The caecal sample (>50 mg) was mixed using 25% metaphosphoric acid and was left standing 30 min, and then it was centrifuged at 4 °C (12,000× *g*, 20 min). The supernatant was purified with a 0.22 μm water filter and transferred to a 300 μL glass micro-insert into a bottle. Analysis was performed using UPLC (Shimadzu, Japan) and incubated as follows: InertSustain AQ-C18 Column (150 mm × 2.1 mm, 1.9 um particle size, the column temperature: 40 °C, Shimadzu, Shimane, Japan); the mobile phase consisted of 20 mmol/L NaH_2_PO_4_ (A, pH 2.2) and acetonitrile (B). The elution procedure was linear gradient of 95% A over 5 min, 80% A for 5 min, and finally 20% A for 10 min. The flow rate was set at 500 uL/min, and injection volume was 10 uL [18].

### 2.10. Statistical Analysis

By using the GraphPad Prism software (version 8.2.1 Windows version, GraphPad Software, San Diego, CA, USA), we determined the significance among groups using a one-way analysis of variance (ANOVA) with a post hoc Tukey’s test. The data was presented as mean ± standard deviation (SD). A *p* value of <0.05 was considered statistically significant.

## 3. Results and Discussion

### 3.1. Purification of HEP10

As shown in Figure S1, the polysaccharides were obtained from the crude polysaccharides of *H. erinaceus* (HECP), using the Sevage method to remove protein. Compared with HECP, the total sugar content of polysaccharides increased from 69.03% to 89.03%, while the protein content of polysaccharides decreased from 23.73% to 6.23%. A fractional peak was eluted with distilled water after purification with a DEAE-Sepharose fast flow column and Sephadex G-75 column chromatograph (Figure 1A,B).

The single and symmetrical peak in the HPGPC indicated that HEP10 was a homogeneous polysaccharide (Figure 1C). The molecular weight of HEP10 was 9.9 kDa. This result is different from that of the polysaccharide fractions in the fruiting body of *H. erinaceus* with molecular weights of 13 kDa [7], 15 kDa [19], 18 kDa [20], 18.3 kDa [10], and 50 and 30 kDa [21]. The difference might be due to the compositional difference between submerged culture and fruiting bodies of *H. erinaceus*. Moreover, extraction methods and the growth conditions might also account for the difference.

### 3.2. Subsubsection Structural Characterization of HEP10

#### 3.2.1. Monosaccharide Composition

The monosaccharide composition of HEP10 was determined by HPAEC system (Figure S2). HEP10 consisted of 6 monosaccharides, including fucose (0.85%), arabinose (5.72%), galactose (7.11%), glucose (84.36%), mannose (0.91%), and xylose (1.05%). This result is similar to our previous study, in which HECP also had glucose as the biggest proportion [7]. However, no rhamnose was found, but fucose was detected. In contrast, another study identified a novel polysaccharide of *H. erinaceus* (HEPF1), which consisted of fucose, galactose, and glucose with a molar ratio of 1:4:1 [22]. Moreover, other heteropolysaccharides such as HEP-1 were isolated from the fruiting bodies of *H. erinaceus*, which was composed of rhamnose, galactose, and glucose with a ratio of 1.19:3.81:1.00 [20].

#### 3.2.2. FT-IR Spectrum

IR spectrum of HEP10 exhibited characteristic absorption peaks of polysaccharide (Figure 1D). The broad and intense band at 3387.1 cm^−1^ belonged to the stretch vibration of hydroxyl groups [23]. The band at 2918.4 cm^−1^ was attributed to the stretching vibration of C–H bond [24]. The absorption peaks at 1606.7 cm^−1^ and 1396.5 cm^−1^ were the result of the asymmetrical and symmetrical stretching vibration of the carboxyl group, respectively. The three absorption peaks at 1137.9 cm^−1^, 1076.2 cm^−1^, and 1024.1 cm^−1^ indicated the presence of pyranose and furanose [24]. The small absorption peak around 890 cm^−1^ was assigned to the β-configuration of glucosyl units [25]. The absorption peak at 842.8 cm^−1^ indicated the α-type glycosidic linkages in HEP10 [26].

#### 3.2.3. NMR Analysis of HEP10

^1^H NMR and ^13^C NMR spectra of HEP10 are shown in Figure 1E,F. Taking the appropriate literature as a reference [24,25,27], the anomeric proton signals appeared at 5.49, 5.38, 5.30, 5.19, and 5.08 ppm, indicating that there are five α-configuration sugar residues. There was resonance peak overlap between 3.46–4.26 ppm. The other anomeric proton signals might be β configuration (Figure 1E). The signals at 100.2 ppm and 104.6 ppm referred to anomeric carbon signals, which indicated the α configuration and β configuration, respectively (Figure 1F). The HMR data suggest that the glycosidic linkage of HEP10 might be (1→2) and (1→6).

### 3.3. HEP10 Inhibited LPS-Induced Inflammation in RAW264.7 Cells

#### 3.3.1. Effect of HEP10 on Cell Viability and Cytokine Production In Vitro

In Figure S3, LPS (1 μg/mL) or HEP10 (12.5–400 μg/mL) treatment did not significantly affect RAW264.7 cell viability determined by MTT assay. These results displayed that HEP10 has little toxic effect on murine macrophages. NO production of the RAW264.7 cells was markedly induced by LPS. Meanwhile, HEP10 (50–200 μg/mL) treatment suppressed NO production induced by LPS significantly. In addition, HEP10 (50–200 μg/mL) downregulated TNF-α and IL-1β secretion induced by LPS in RAW264.7 cells. Furthermore, HEP10 (25–100 μg/mL) suppressed LPS-induced TNF-α and IL-1β gene expression. Therefore, we chose the concentration between 25–200 μg/mL of HEP10 for subsequent study. 

#### 3.3.2. Effect of HEP10 on LPS-Induced Inflammasome Activation In Vitro

**Figure 2:**
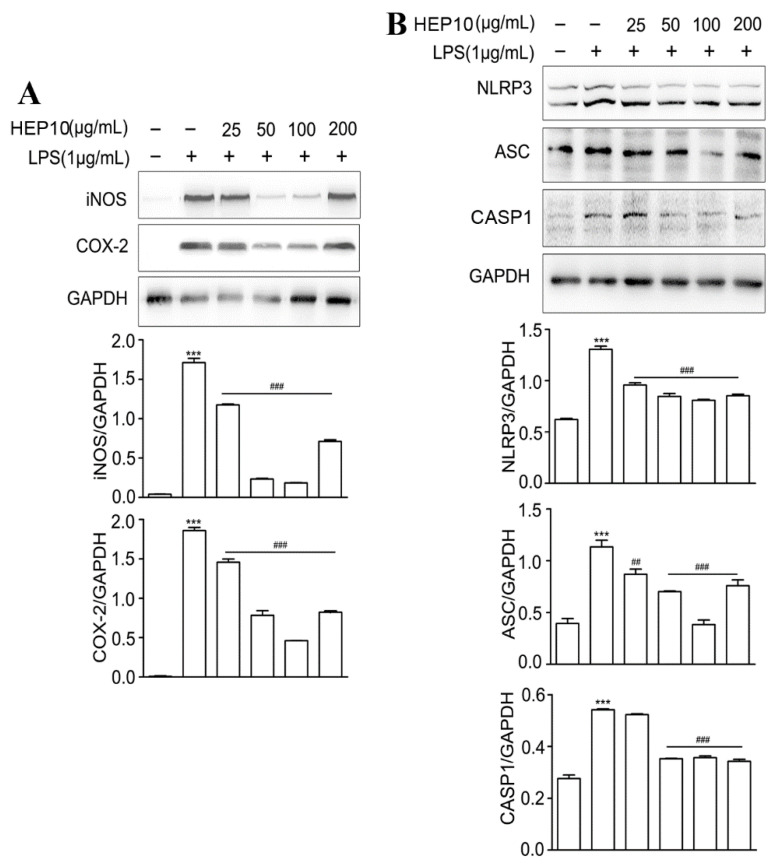
LPS-induced inflammation and NLRP3 inflammasome were downregulated in HEP10-treated RAW264.7 cells. HEP10 (25–200 μg/mL) treated for 1 h and then induced by LPS (1 μg/mL) for 2 h. (**A**,**B**) Western blot analysis of iNOS, COX-2, NLRP3, ASC, and CASP1 production in RAW264.7 cells. The data is presented as means ± SD (*n* = 3), *** *p* < 0.001 vs. the CTL. ^##^ *p* < 0.01 and ^###^ *p* < 0.001 vs. the LPS treatment.

iNOS catalyzes the NO production, and COX-2 catalyzes the prostaglandin production, which both reflect the degree of inflammation [28]. They are mainly synthesized by inflammatory cells [29] and help build up the inflammatory environment, which causes intestinal damage in UC [30]. When compared to the CTL group, there were higher protein levels of iNOS and COX-2 after LPS stimulation (Figure 2A). INOS and COX-2 expression were significantly inhibited by HEP10 (25–200 ng/mL) treatment (Figure 2A). Notably, when the concentration of HEP10 was 25–100 μg/mL, its inhibitory effect on iNOS and COX-2 was dose-dependent, while when the concentration was greater than 100 μg/mL, its inhibitory effect on iNOS and COX-2 was weakened.

The activation of NLRP3 inflammasomes results in the activation of CASP1 and subsequent cleavage of proinflammatory cytokines IL-1β [3]. Productions of IL-1β, NLRP3, ASC, and CASP1 indicate the activation of NLRP3 inflammasome [4]. LPS challenge significantly upregulated the expression of IL-1β, NLRP3, ASC, and CASP1(Figure S3), whereas HEP10 (50–200 μg/mL) significantly downregulated their protein expressions (Figure S3 and Figure 2B). Remarkably, the dose-dependent regulation of NLRP3 and ASC was observed within the HEP10 concentration range of 25–100 μg/mL. When the concentration was greater than 100 μg/mL, the inhibition of NLRP3 and ASC was weaker than that of 100 μg/mL HEP10. These results suggest that HEP10 (25–100 μg/mL) could inhibit NLRP3 inflammasome activation by suppressing the production of the abovementioned inflammasome components.

#### 3.3.3. HEP10 Downregulated LPS-Induced NF-κB, AKT, and MAPK Signaling In Vitro

**Figure 3:**
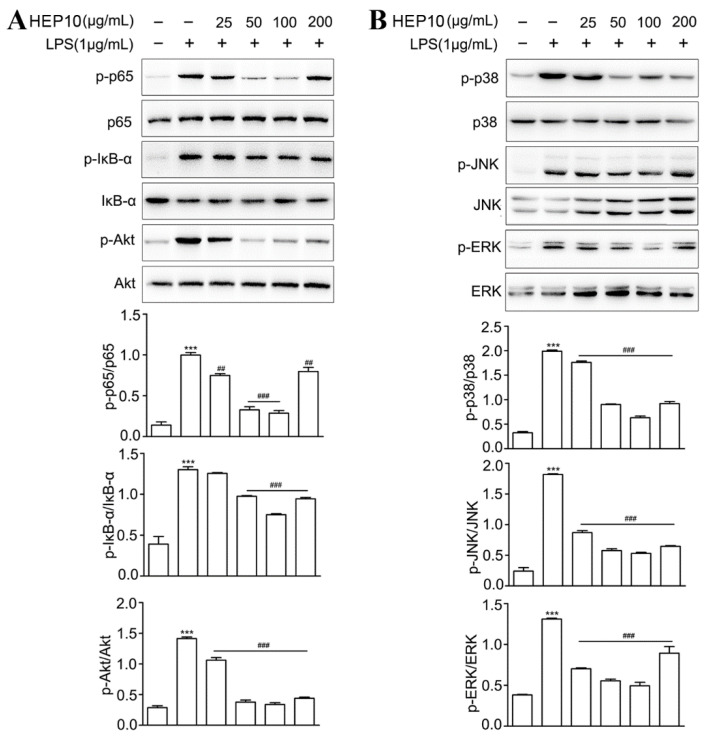
HEP10 inhibited NF-κB, Akt, and MAPK signaling in LPS-induced RAW264.7 cells. HEP10 (25−200 μg/mL) treated for 2 h and followed by LPS (1 μg/mL) stimulation for 24 h. Total cellular extracts were analyzed by western blotting to determine the levels of (**A**) phosphorylated p65, IκB, Akt, (**B**) P38, JNK, and ERK. The membranes were re-probed with antibodies against total p65, IκB, Akt, P38, JNK, and ERK for normalization. The data is presented as means ± SD (*n* = 3), *** *p* < 0.001 vs. the CTL group. ^##^ *p* < 0.01 and ^###^ *p* < 0.001 vs. the LPS treatment.

NF-κB and AKT signaling pathways regulate gene transcription, protein synthesis, and cytokine production in macrophages [31,32]. As shown in Figure 3, there were higher protein levels of p-p65, IκB-α, and Akt after LPS stimulation compared with the CTL group. HEP10 (50–200 μg/mL) treatment significantly inhibited LPS-induced p-p65, p-IκB, and p-Akt expression. The ERK, JNK, and p38 are conventional mitogen-activated protein kinases (MAPKs) that participate in a number of fundamental cellular processes such as growth, differentiation, apoptosis, and inflammation [33]. Western blot analysis showed that HEP10 (50–200 μg/mL) significantly downregulated LPS-induced p-p38, p-JNK, and p-ERK expression levels. Notably, similar to the previous results, HEP10 with a concentration of 200 g/mL showed a lower effect than that of 100 g/mL. These results together imply that HEP10 has a potent anti-inflammatory role in LPS-stimulated RAW cells by inhibiting NF-κB, AKT, and MAPK signaling.

### 3.4. Anti-Inflammatory Effects of HEP10 in Mice with Ulcerative Colitis

#### 3.4.1. HEP10 Attenuated the Severity of Colitis in Mice Treated with DSS

**Figure 4:**
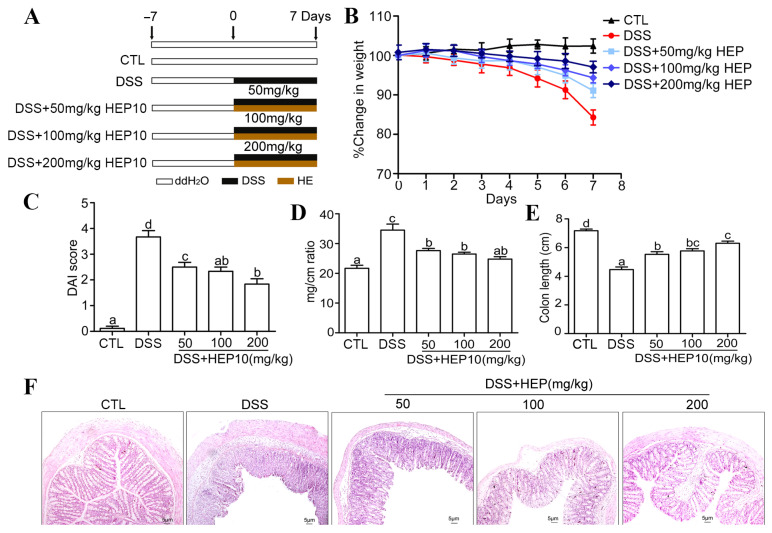
Administration of HEP10 attenuated mice’s colitis induced by 2% DSS. (**A**) Experimental protocol for the treatment of HEP in DSS-induced colitis in male C57BL/6J mice (*n* = 5 per group). (**B**) The changes of body weight during the disease progression. (**C**) DAI score, (**D**) colon mg/cm ratio, (**E**) colon length, and (**F**) representative image of H&E staining of colons tissue from each group. Scale bar: 5 μm. The data is presented as means ± SD (*n* = 5). Different alphabetic letters refer to significant differences (*p* < 0.05, two sided).

To evaluate the anti-inflammatory effects of HEP10 in vivo, a murine colitis model induced by DSS was used [15]. As expected, the mice showed body weight loss after drinking 2% DSS for 7 days (Figure 4A,B). Treatment with HEP10 (50–200 mg/kg) ameliorated body weight loss compared with DSS group (Figure 4B). There was a significant upregulation of DAI score in the DSS group compared with the CTL group, indicating the severity of inflammation with DSS administration (Figure 4C). The effect of HEP10 treatment was dose-dependent, which was reflected by reduced incidence of diarrhea and blood in feces and decreased DAI score (Figure 4C). Shortening of the colon and an increment of weight/length ratio also reflected the severity of colitis in DSS group, which were reversed with HEP10 treatment (Figure 4D,E). Moreover, HEP10 treatment significantly attenuated the histological symptoms of epithelial disruption and colonic mucosal ulcers caused by DSS administration (Figure 4F). These data suggest that HEP10 could significantly ameliorate the clinical symptoms of colitis induced by DSS in mice.

#### 3.4.2. HEP10 Inhibited Oxidative Stress and Cytokine Production in Colon Tissues of Mice with DSS-Induced Colitis

**Figure 5:**
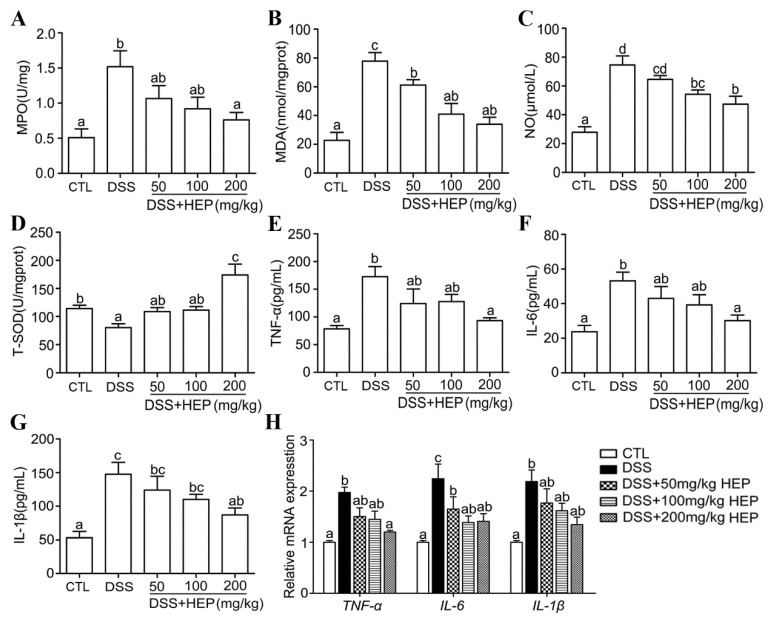
HEP10 inhibited inflammatory and oxidative damage on mucosa in C57BL/6 mice with DSS-induced colitis. (**A**) MPO level, (**B**) MDA level, (**C**) NO level, (**D**) T-SOD activity, (**E**) TNF-α, (**F**) IL-1β, and (**G**) IL-6 levels in colonic content were determined by ELISA. (**H**) Relative gene expression of TNF-α, IL-1β, and IL-6 were detected by qRT-PCR. The data is presented as means ± SD (*n* = 5). Different alphabetic letters refer to significant differences (*p* < 0.05, two sided).

MPO is an indicator of neutrophil infiltration and leads to mucosal disruption in UC [34]. The results showed that the MPO activity in colon tissues from the DSS group was significantly upregulated compared with CTL group (Figure 5A), which confirmed the tissue injury and inflammation in the DSS group. HEP10 administration exhibited a significant inhibitory activity against DSS-induced MPO activity (Figure 5A).

Oxidative stress is a major cause and the therapeutic target for UC [35]. The excessive production of malondialdehyde (MDA) and NO cause oxidative damages [36]. We observed that the MDA and NO production in the DSS group were significantly enhanced compared to that of the CTL group. Meanwhile, the expression of antioxidant enzyme T-SOD was lower than that of the CTL group (Figure 5B–D). The administration of HEP10 dose dependently reversed DSS-induced changes in NO concentration, MDA, and T-SOD levels (Figure 5B–D).

Inflammatory cytokines, which play an important role in both innate and adaptive immunity, also contribute to the pathogenesis of UC [37]. Higher levels of inflammatory cytokines such as IL-1β, IL-6, and TNF-α, which can cause damage in colon tissues, were detected in the DSS group as expected (Figure 5E–G). Treatment with HEP10 significantly suppressed IL-1β, IL-6, and TNF-α production in colon tissues from DSS-induced colitis mice (Figure 5E–G). Furthermore, HEP10 attenuated gene expression levels of TNF-α, IL-6, and IL-1β (Figure 5H). These data indicate that HEP10 might play a role in reducing the production of inflammatory cytokines and lowering the oxidative stress in the colonic mucosa to achieve the remedial effect against UC.

#### 3.4.3. HEP10 Blocked Activation of NLRP3 Inflammasome in Colon of Mice with DSS-Induced Colitis

**Figure 6:**
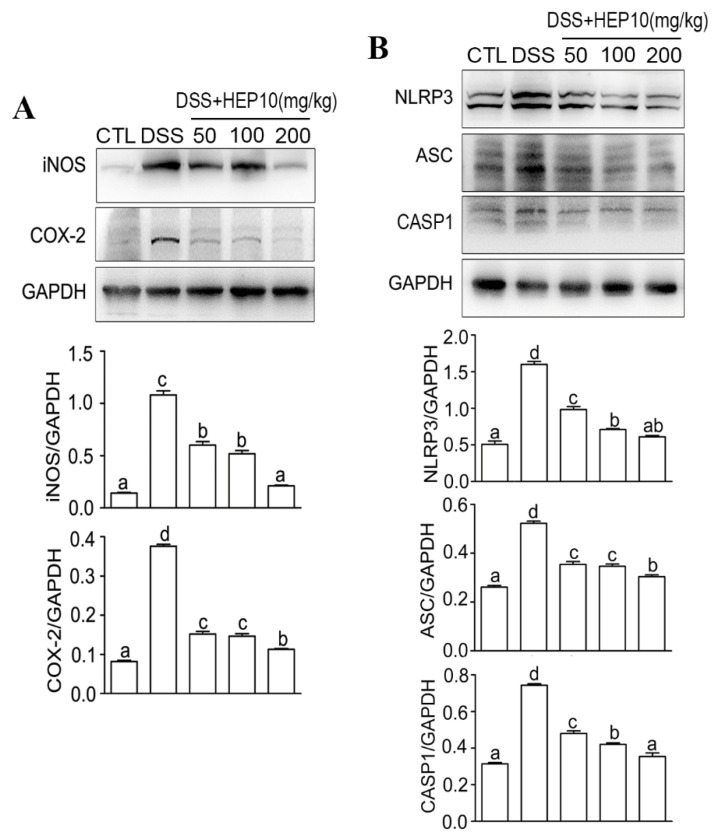
HEP10 inhibited the expression of iNOS, COX-2, and NLRP3 inflammasome in colon tissue of C57BL/6 mice with DSS-induced colitis. Western blot analysis of (**A**) iNOS, COX-2, (**B**) NLRP3, ASC, and CASP1 production. The data is presented as means ± SD (*n* = 5). Different alphabetic letters refer to significant differences (*p* < 0.05, two sided).

Inhibiting the inducible enzymes iNOS and COX-2 synthesized by inflammatory cells could prevent tissue injury [29]. The higher expression levels of iNOS and COX-2 in the DSS group than in the CTL group confirmed the presence of inflammation in colon tissues (Figure 6A). Treatment with HEP10 significantly decreased the iNOS and COX-2 expression induced by DSS in mice (Figure 6A). The NLRP3 inflammasome has been proven to play a critical role in intestinal inflammation in DSS-induced colitis [5,6]. Consistently, the upregulation of protein expressions of NLRP3, ASC, and CASP1 in our experiment demonstrated that DSS activated the NLRP3 inflammasome in colon tissues (Figure 6B). Notably, HEP10 inhibited the expression of NLRP3 inflammasome in colitis mice (Figure 6B). Same as the previous result in RAW264.7 cells, HEP10 also significantly reduced the mRNA and protein expression levels of IL-1β in the colon tissues of DSS-treated mice (Figure 5G,H). According to these findings, HEP10 might attenuate DSS-induced colitis through inhibiting NLRP3 activation.

#### 3.4.4. HEP10 Blocked NF-κB, AKT, and MAPK Signaling in Colon of Mice with DSS-Induced Colitis

**Figure 7:**
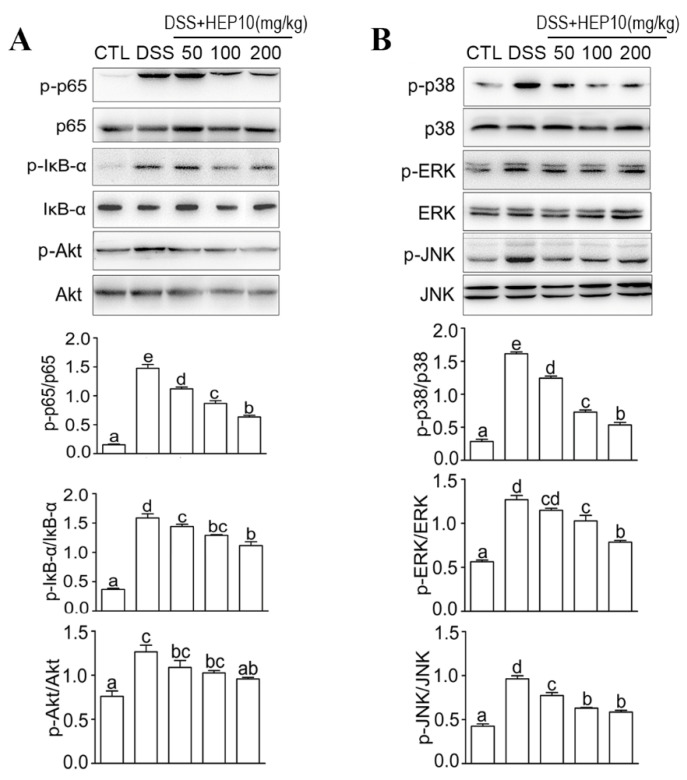
HEP10 inhibited NF-κB, Akt, and MAPK signaling in colon tissues of mice with DSS-induced colitis. Protein expression levels of phosphorylated p65, IκB, and Akt determined by Western blotting (**A**), P38, ERK, and JNK (**B**). The membranes were re-probed with antibodies against total p65, IκB, Akt, P38, ERK, and JNK for normalization. The data is presented as means ± SD (*n* = 5). Different alphabetic letters refer to significant differences (*p* < 0.05, two sided).

The NF-κB, AKT, and MAPK signaling pathways regulate the expression of inflammatory cytokines such as iNOS, COX-2, TNF-α, IL-6, and IL-1β. They are also involved in modulating oxidative stress and inflammasome activation in UC [4,35]. The phosphorylation of p65, IκB, AKT, and MAPK was increased in colon tissues of mice treated with DSS compared with the CTL group. As expected, HEP10 inhibited the phosphorylation of the abovementioned signaling molecules in colon tissues of mice treated with DSS (Figure 7). This effect further contributed to the reduction in NO, inflammatory cytokine production, and inflammasome activation under DSS-induced UC. These data indicate that HEP10 ameliorates DSS-induced colitis in mice by inhibiting NF-κB/Akt/MAPK signaling.

### 3.5. HEP10 Modulated the Structure and Metabolic Function of the Gut Microbiota in Mice

**Figure 8:**
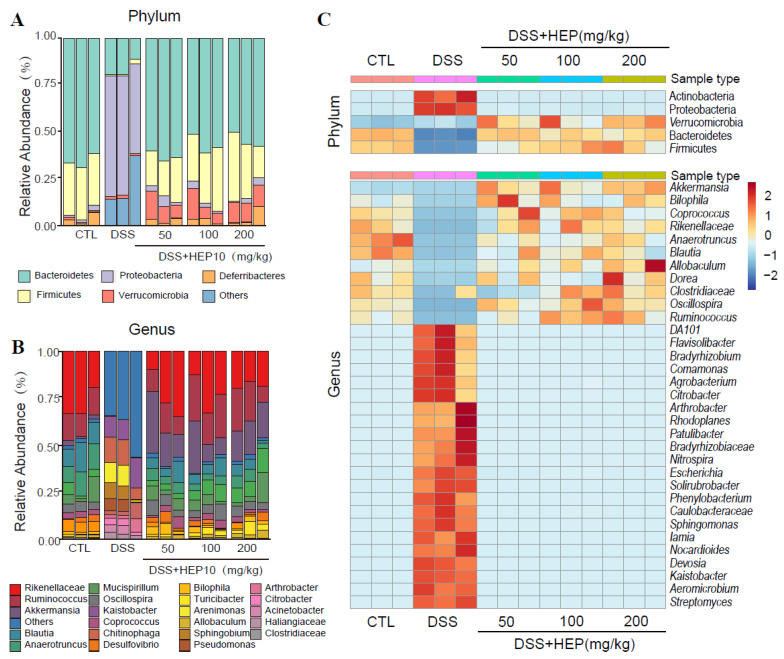
Comparisons of gut microbial compositions. (**A**,**B**) Compositions at the phylum and genus levels. (**C**) The heatmap shows the abundance of top 5 phyla and top 33 genera altered by HEP10 in DSS-induced mice model.

Caecal content samples were sequenced for 16S rDNA to determine whether HEP10 regulates gut microbiota. As shown in Figure S4, alpha diversity and beta diversity suggested that HEP10 restored the original richness of gut microbiota. As shown in Figure 8, the most abundant phylums were Firmicutes and Bacteroidetes. Comparing DSS treatment with CTL treatment, Proteobacteria and Actinobacteria were upregulated, while Firmicutes and Bacteroidetes were downregulated. HEP10 (50–200 mg/kg) could reverse these changes by decreasing the relative abundance of the phylum Proteobacteria, while increasing *Akkermansia muciniphila*. Further analysis of operational taxonomic units (OTUs) found that different doses of HEP10 groups reversed the change of gut microbiota to different degrees in mice with DSS-induced colitis, among which 200 mg/kg HEP10 had significant effect on 47% of key OTUs (*p* < 0.05), while the ratio of the 50 mg/kg HEP10 group was 31% (Figure S5). This suggested that the high-dose HEP10 group (200 mg/kg) may have better modulatory effects on gut microbiota.

In Figure S6, we used PICRUSt to predict functions of KEGG categories represented in mice treated with 200 mg/kg HEP10. There were 11 differentially abundant KEGG metabolic pathways between the CTL and DSS groups (*p* < 0.01, 5 pathways increased and 6 pathways decreased), including the amino acid metabolism- and carbohydrate metabolism-related microbial genes. The abundance of 10 metabolic pathways was reversed in the HEP10 group compared with the DSS group after 200 mg/kg HEP10 treatment. Notably, it was apparent that the amino acid metabolism pathway (Figure S6B) and carbohydrate metabolism pathway (Figure S6C) of the 200 mg/kg HEP10 group were reversed. We also examined changes in SCFAs in ceacal contents. SCFAs are considered key players in target multiplication in host localities and systems, including metabolic and immune processes. As shown in Figure S6D, after HEP10 intervention, the content of fecal short chain fatty acids recovered significantly.

**Figure 9:**
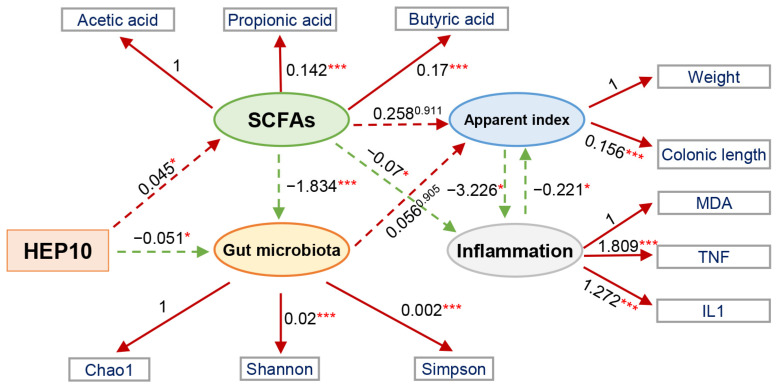
The structural equation modeling showed the indirect (dashed arrows) effects and direct (continuous arrows) effects of HEP10 on an apparent index, the abundance and diversity of bacteria, SCFAs, and inflammatory markers. Positive and negative relationships are indicated by red and green arrows, respectively. Significance levels are: * p < 0.05; *** p < 0.001.

Furthermore, we developed a structural equation model to investigate the contribution of HEP10 to the apparent index, inflammatory markers, and microbial communities (Figure 9). The structural equation model is a statistical method that analyzes the relationship between variables by using covariance matrixes of variables. It is an important tool for analyzing multivariate data analysis. It perfectly combines the measurement equation with the structural equation and is one of the important means of multivariate data analysis [17,38]. Nine latent-manifest variables were linked in order to explore their significance, and five of these showed statistically significant relationships (*p* < 0.05). There was a positive correlation between HEP10 intervention and SCFAs (−0.045, *p* < 0.001) but a negative correlation between HEP10 intervention and diversity of bacteria. This may be related to the fact that HEP10 can significantly decrease the relative abundance of the phylum Proteobacteria. The results of our study show that HEP10 can change widely through gut microbiota.

## 4. Conclusions

In this study, we isolated and purified a polysaccharide HEP10 from the crude polysaccharides of *H. erinaceus* with an average molecular weight of 9.9 kDa. HEP10 consisted of glucose, galactose, rhamnose, xylose, amino-galactose, mannose, fucose, and glucosamine, which may involve both α and β glycosidic linkages. We further investigated the effect of HEP10 on LPS-challenged murine macrophage RAW264.7 cells and in C57BL/6 mice with DSS-induced colitis. The results revealed that HEP10 treatment suppressing LPS/DSS-stimulated production of NO, COX-2, iNOS, and inflammatory cytokines including TNF-α, IL-1β, and IL-6. HEP10 also blocked LPS/DSS-induced NLRP3 inflammasome activation. Moreover, HEP10 inhibited NF-kB p65, Akt, and MAPK phosphorylation, which were elevated by LPS/DSS. Our data demonstrated that HEP10 possesses potential therapeutic capability to treat experimentally induced acute UC in mice via inhibiting production of inflammatory cytokines, NLRP3 inflammasome activation, and phosphorylation of NF-κB/AKT/MAPK. HEP10 could reverse almost all varieties of DSS-enhanced bacterial species, especially *Akkermansia muciniphila*, and also promote functional shifts in gut microbiota. HEP10 has a better prebiotic effect than the crude polysaccharides of *H. erinaceus*, which can be used as a novel dietary supplement and prebiotic to ameliorate colitis.

## Data Availability

The raw data of 16S rRNA high-throughput sequencing was uploaded to the Sequence Read Archive (SRA) database of NCBI (PRJNA679399). Other data will be made available by the authors, without undue reservation.

## Supplementary Materials

The following supporting information can be downloaded at: https://www.mdpi.com/article/10.3390/nu15030739/s1, Figure S1: Purification flowchart of HEP10 from the crude polysaccharides of *H. erinaceus*. Figure S2: Chromatogram of standard monosaccharides and monosaccharides of HEP10. Figure S3: Effects of HEP10 on cell viability, NO, and cytokine production in LPS-treated RAW264.7 cells. Figure S4: Metagenomic analysis of microbiota in the caecum content at the diversity. Alpha diversity analysis and beta diversity analysis. Figure S5: Heatmap showing the abundance of top 100 OTUs altered by HEP10 in DSS-fed mice based on RDA. Figure S6: Prediction of metabolic function and the concentrations of SCFAs in the DSS-induced mice after HEP10 (200 mg/kg) treatment.
