# Supplementary material for: Low Weight Polysaccharide of Hericium erinaceus Ameliorates Colitis via Inhibiting the NLRP3 Inflammasome Activation in Association with Gut Microbiota Modulation
Source: Nutrients. 2023 Feb 1;15(3):739. doi: 10.3390/nu15030739 (PMC9920828; doi:10.3390/nu15030739)
Supplement: Supplementary file 1 [file nutrients-15-00739-s001.zip › nutrients-2082929-supplementary.pdf]

Supplementary Materials

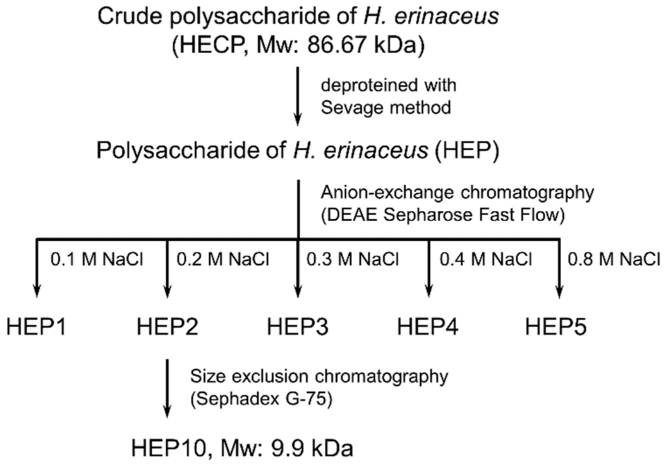

Figure S1. Purification flowchart of HEP10 from the crude polysaccharides of *H. erinaceus*.

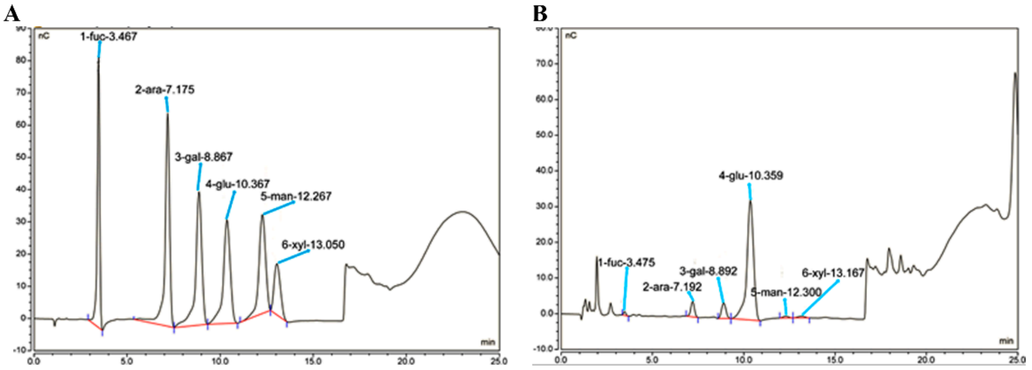

Figure S2. Chromatogram of standard monosaccharides (A) and monosaccharides of HEP10 (B). 1: fucose; 2: arabinose; 3: galactose; 4: glucose; 5: mannose; 6: xylose.

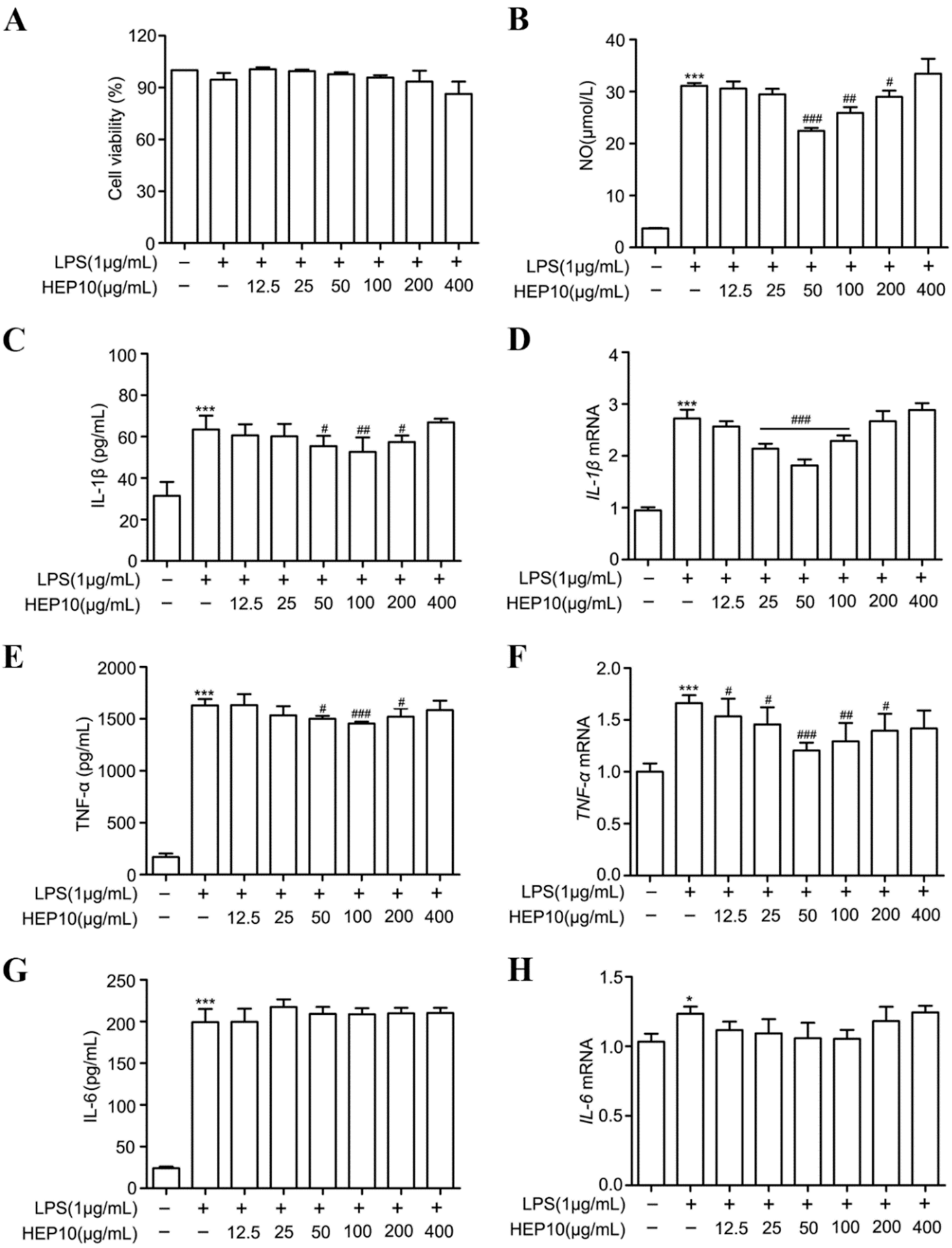

Figure S3. Effects of HEP10 on cell viability, NO and cytokine production in LPS treated RAW264.7 cells. RAW264.7 cells were pre-treated with HEP10 (25-200 μg/mL) for 2 h and then induced by LPS (1 μg/mL) for 24h. Cell supernatant was used for NO detection or ELISA. Total cell RNA was used for qRT-PCR analysis. (A) Cell viability determined by MTT assay. Different concentrations of HEP prevent the production of LPS induced NO (B) and inflammatory cytokines IL-1β (C, D), IL-6 (E, F) and TNF-α (G, H) in RAW264.7 cells. Data represent means ± SD (n = 3), ****P < 0.001* vs the CTL group. #*P < 0.05*, ##*P < 0.01* and ###*P < 0.001* vs the LPS treatment.

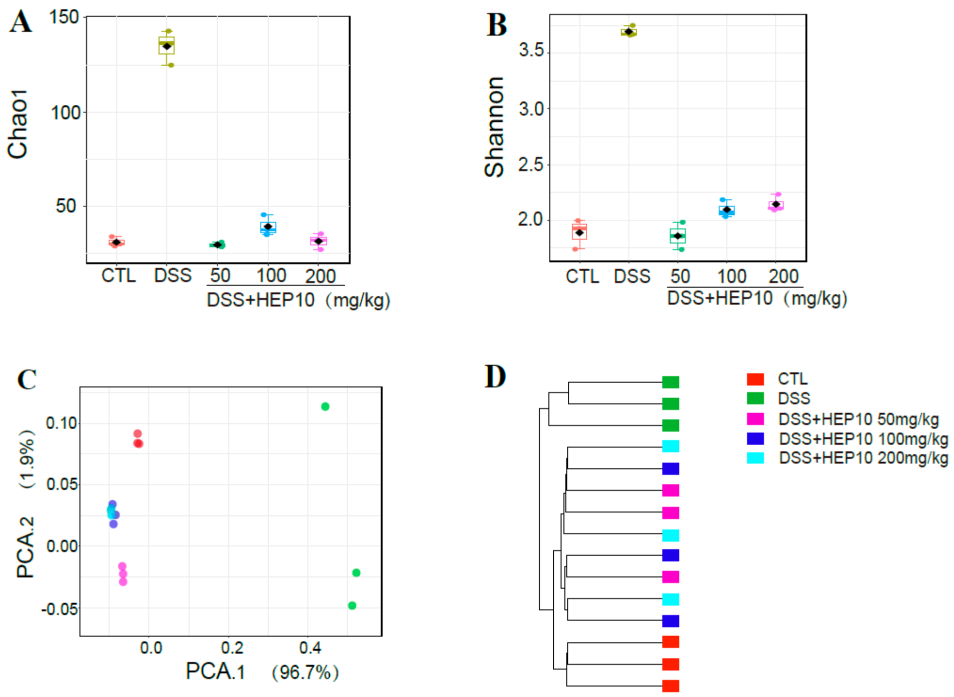

Figure S4. Metagenomic analysis of microbiota in the caecum content at the diversity. Alpha diversity analysis (A, B) and beta diversity analysis (C, D).

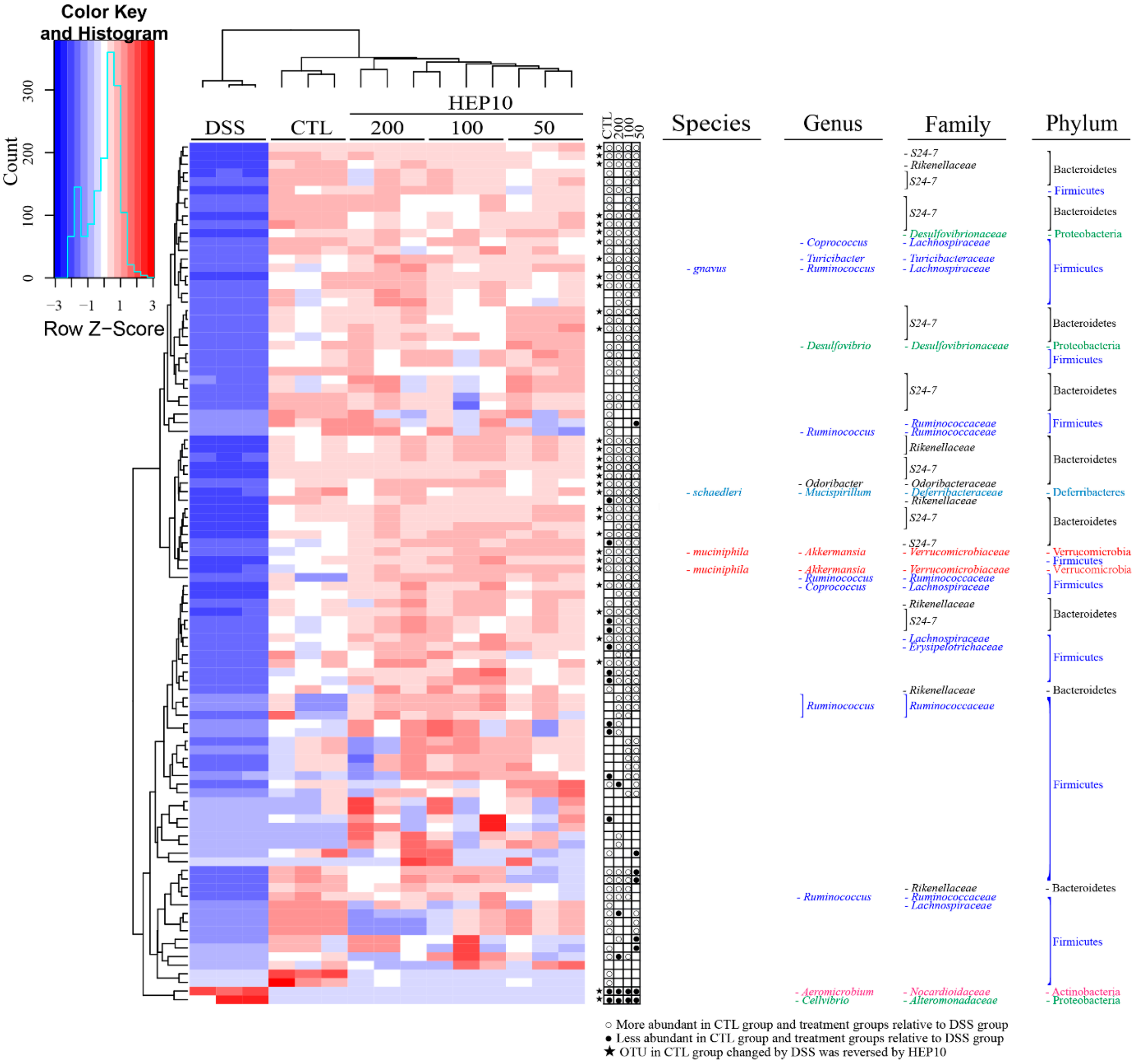

Figure S5. Heatmap showing the abundance of top 100 OTUs altered by HEP10 in DSS-fed mice based on RDA. The color of the spot corresponds to the normalized and log-transformed relative abundance of the OTUs. The OTUs are organized according to their phylogenetic positions. The taxonomic assignment of each OTU was determined by RDP classifier and the genus name was labeled in the graph. OTU taxonomy is shown on the left. Represented bacterial taxa information (species, genus, family and phylum) of 100 OTUs are shown on the left.

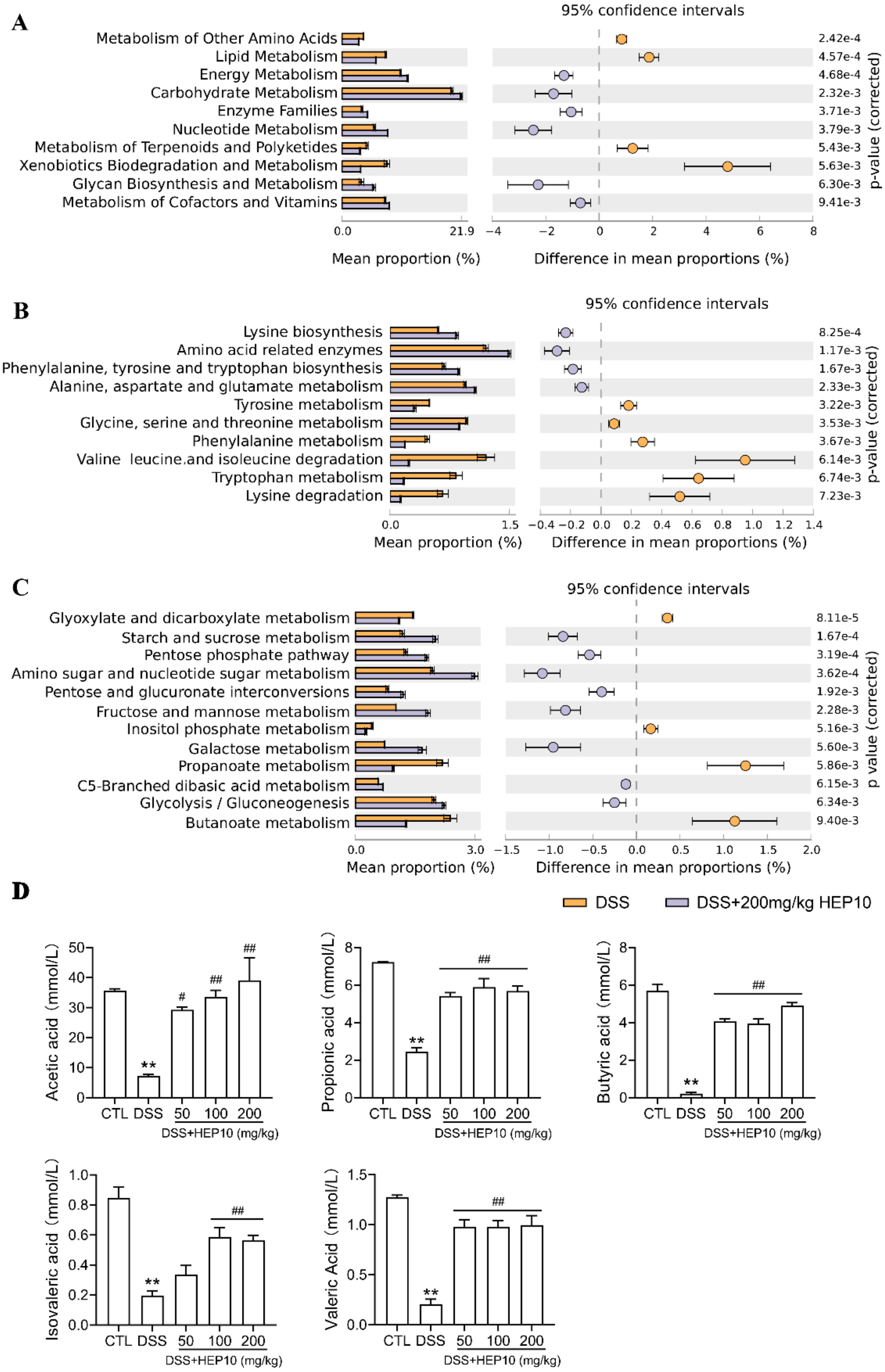

Figure S6. Prediction of metabolic function and the concentrations of SCFAs in the DSS-induced mice after HEP10 (200 mg/kg) treatment. Metabolism pathway(A), amino acid metabolism pathway and carbohydrate metabolism pathway (C) in caecal content microbiomes. (D) The concentrations of acetic acid, propionic acid, butyric acid, isovaleric acid, and valeric acid in the colonic contents of mice. Data represent means ± SEM (n =5). Compared with control group (CTL), $^{**}P < 0.01$; compared with DSS induced group (DSS), $^{\#}P < 0.05$, $^{\#\#}P < 0.01$.
